# Influence of road and traffic conditions on emissions and fuel consumption of light vehicles in a real urban driving cycle

**DOI:** 10.1007/s11356-025-36573-3

**Published:** 2025-06-05

**Authors:** Constantin Nitoiu, Corneliu Cofaru, Mihaela Popescu

**Affiliations:** Faculty of Mechanical Engineering, Transilvania University of Brașov, Street Erolilor 29, 500030 Brașov, Romania

**Keywords:** Real traffic, Traffic intensity, Pollutant emissions, Fuel consumption, Dynamic cycles

## Abstract

Over time, international decision-making bodies have paid a special attention to the adoption of test driving cycles in the laboratory that reflect standard traffic conditions and regulations regarding the maximum limit of pollutant emissions from road vehicles to be homologated for their admittance to traffic. The proposed study examines the impact of road and traffic characteristics on pollutant emissions and fuel consumption of light vehicles in a real urban traffic cycle. The research was carried out following two scenarios, in two phases, each of them focusing on the analysis of greenhouse gas emissions mainly (CO_2_), nitrogen oxides (NO_x_), and carbon monoxide (CO) from motor vehicles with both petrol and diesel. Simultaneously, the fuel consumption of these vehicles was also evaluated. In the first scenario, the objective was to collect real data on road and traffic characteristics through tests on a carefully selected urban route in two phases: noon (light traffic) and evening (congested traffic). In the second scenario, data from both phases were used to generate two driving cycles simulating real conditions for vehicle testing on the stand; thus, four different cars were used. The research results obtained both by traveling the chosen route and by virtually traveling the same route by using real driving cycles allow a complex analysis of the interaction of different types of cars (Standard EURO 6) with the surrounding environment in traffic conditions that can lead to improve substantial effects of impacts on its various components.

## Introduction

Transport activities assume an important share of the total energy consumption. In 2022, this consumption represented 31.0% of the final energy consumption in the EU.

Road transport, which includes urban transport in the same period, represented 73.6% of all energy consumed in the transport sector in the EU, much higher than air transport (11.4%), water transport (13.0%), and transport railway (1.4%).

Conventional fuels used in road transport in the EU, such as petrol, gas, and diesel, accounted for 90.6% of energy consumption (Eurostat 2024).

At the same time, the road transport sector is also assigned important quotas regarding gaseous pollutant emissions and solid particles (European Environment Agency 2024).

At the same time, it should be stated that vehicle emissions, fuel consumption, and road and traffic characteristics, as well as the driver’s driving style, are interconnected elements that define the impact and the behavior of vehicles in the urban environment (Fangfang et al. 2017; Julie et al. 2024; Yichen et al. 2022; Vladimir et al. 2023). The pollution standards imposed on vehicles and the technologies applied to vehicles directly influence the level of vehicle emissions and fuel consumption (Lie et al. 2022; Roberts et al. 2014; Tarulescu et al. 2016).

The urban road network and its characteristics impose traffic control systems that generate decelerations, stops, and accelerations of moving vehicles and that can determine congested traffic conditions where the speed of travel is drastically reduced and stops are frequent (Evangelos et al. 2018). This phenomenon, known as road congestion, can increase fuel consumption and, implicitly, pollutant emissions due to low traffic speeds if the start & stop function is not present on board the vehicles (although on most new vehicle models it is present, it comes into operation when the thermal regime reaches a certain minimum temperature).

In general, fuel consumption and vehicle emissions depend on traffic conditions and driver’s and vehicle’s characteristics, especially the propulsion system (Ibrahim et al. 2022; Kumar et al. 2011).

Numerous studies and experimental research have shown that car engines work inefficiently in urban traffic due to the low speed and load regimes on the one hand and, on the other hand, the low thermal regime of the engine, leading to inefficient combustion of fuel and generating polluting emissions, especially at peak hours when traffic jams are inevitable (Suresh et al. 2013). The amount of fuel a vehicle burns to generate power and the move is directly related to its emissions. The more fuel a vehicle consumes, the more pollutants it emits into the atmosphere. These emissions include greenhouse gases (such as carbon dioxide (CO_2_)), gaseous pollutants (carbon monoxide (CO), nitrogen oxides (NO_x_), hydrocarbons (HC)), solid pollutants, fine particles (PM2.5), and other harmful substances from other vehicle systems (e.g., brakes). These emissions can affect air quality, human health, flora and fauna, buildings, and the environment (Attfield et al. 2011; Ioannis et al. 2020; Silverman et al. 2012). The main efforts made in this field were made on urban traffic because, in these areas, the highest values of polluting emissions are generated, being directly influenced by the growing number of vehicles and variable travel speeds (acceleration/deceleration) (Ahn and Rakha 2009; De Vlieger et al. 2002; Lv and Zhang 2012; Ma et al. 2015; Qu et al. 2015; Zhang et al. 2011).

The research program pursued three objectives:

- To obtain real fuel consumption and emissions data for a specific urban traffic
- To obtain a relationship between consumption and emissions on the one hand and traffic flow characteristics on the other hand
- To collect data for a traffic simulation tool designed to test vehicles to reduce fuel consumption and pollutant emission levels

The analysis of the test results must take into account the fact that the operating regimes of the vehicles chosen during the tests have a dynamic character that develops a wide variety of physical and chemical mechanisms at the engine level (Hasan et al. 2022).

These mechanisms are manifested in the processes of gas exchange, the formation of air–fuel mixtures, and the combustion process, which are influenced by the energy level at which they take place, the result being positive or negative on emissions and fuel consumption (Haiyong et al. 2008; Heywood 2018).

Vehicles that comply with the EURO 6 standard, equipped with both gasoline and diesel engines, are equipped with equipment to reduce fuel consumption and thereby reduce CO_2_ emissions and devices to reduce pollutant emissions.

It should be noted that the vehicles chosen for the tests have different exhaust gas treatment systems that depend on the type of engine. In the case of gasoline engines, the three-way catalytic converter (TWC) is used because it uses a homogeneous air–fuel mixture of stoichiometric quality (λ = 1). In this converter, the oxidation of unburned hydrocarbons and carbon monoxide and the reduction of nitrogen oxides are carried out; the conversion rate of these pollutants is obtained for the exhaust gases resulting from the combustion of stoichiometric mixtures that have a temperature higher than the catalytic material’s entry into function (catalyst light-off).

Instead, diesel engines that use lean air–fuel mixtures (λ** > **1) are equipped with two treatment systems, one to oxidize unburned hydrocarbons and carbon monoxide and a separate system to reduce nitrogen oxides. This system needs a reductant to activate the catalytic material to the NO_x_ molecules by removing the excess oxygen that tends to block their reduction reactions.

This is the selective reduction system (SCR), the reductant being urea from which ammonia results in the system and which contributes to the reduction of nitrogen oxides. Recent studies have shown that NO_x_ conversion efficiency is highly dependent on exhaust gas temperature. As the temperature increases, the conversion rate first increases and then decreases. NO_x_ reduction reactions generally start at 200 °C and a maximum conversion rate is reached at 350–400 °C. If the temperature of the exhaust gases is lower than 200 °C, the urea decomposition reaction can generate by-products in the form of deposits on the active wall of the SCR converter, which are difficult to remove and which reduce its conversion efficiency. To avoid this, urea injection into the exhaust gas stream will generally start when the gas temperature is greater than 200 °C. Other factors that can influence the efficiency of NO_x_ conversion can be mentioned: the structure of the SCR reactor, the parameters of the catalyst layer, the type of catalyst, the reaction temperature, the volume of the combustion gases, the spatial velocity of their flow, and finally the NH3 molar ratio/NO (Muhamad and Ocktaek 2022; Xin et al. 2019).

Both types of engines used on the test vehicles are equipped with exhaust gas recirculation (EGR) systems to reduce the rate of formation of nitrogen oxides during processes in the engine cylinders. The valve for regulating the flow of recirculated burnt gases is made by each manufacturer for a certain thermal regime of the engine, and also taking into account other factors, such as atmospheric temperature, fuel characteristics, compression ratio, and the shape of the combustion chamber (Haiyong et al. 2008).

## The mathematical model

In this scientific paper, the mathematical model represents a formal and structured representation of the relationships and interactions between the variables and phenomena analyzed during real road tests. This model is a description with the help of mathematical equations, formulas, or algorithms (AutoCad) that reproduce the behavior or the real phenomenon in a simplified and systematic way as precisely as possible, thus giving the possibility of repeating the tests in specially equipped laboratories, thus reaching reliable and accurate conclusions.

The present model allows the simulation and predictability of the behavior or evolution of a road vehicle under various conditions or scenarios, without the need to carry out expensive experiments or real-time tests each time, offering the possibility to test hypotheses and theories in a rigorous and structured way, comparing model results with observed data from experiments or empirical observations. By analyzing and using this mathematical model, optimal solutions can be identified or predictions can be made, thus creating other optimal work scenarios.

During the tests in real traffic, data were recorded regarding several parameters that have a major influence on pollutant emissions, such as travel speed, calculated load value, ambient temperature and engine thermal regime, engine speed, and fuel consumption (Grote et al. 2016; Hamidreza et al. 2023; Yuhan et al. 2021).

By inserting data (collected from real traffic) into the software of the Maha LPS 3000 dynamometric stand, it is possible to generate the help of the “Driving Cycle” option, a series of urban test cycles that can simulate real conditions to test other vehicles on the test stand.

The tests on the stand will follow the traffic conditions recorded, the operation of each tested vehicle under the conditions in which the data specific to each one is loaded, such as the weight of the vehicle, the technical data regarding the frontal surface of the vehicle, and the loading coefficient of the road (slope/ramp.

In the following rows, the main coefficients used by the dynamometric stand for each vehicle studied will be calculated (Tables 1).

**Table 1:** The formulas used to calculate the coefficients of the Maha LPS 3000 dynamic stand (LPS 3000 Dynamometer for Passengers Cars)

Value of traction load (constant)	F = Cf A + $\frac{{\varvec{C}}{\varvec{f}}{\varvec{B}}\bullet {\varvec{v}}}{{{\varvec{v}}}_{{\varvec{r}}{\varvec{e}}{\varvec{f}}}}+\frac{{\varvec{C}}{\varvec{f}}{\varvec{C}}\bullet {{\varvec{v}}}^{2}}{{{\varvec{v}}}_{{\varvec{r}}{\varvec{e}}{\varvec{f}}}^{2}}+\frac{{\varvec{C}}{\varvec{f}}{\varvec{D}}\bullet {{\varvec{v}}}^{\mathbf{exp}{\varvec{D}}}}{{{\varvec{v}}}_{{\varvec{r}}{\varvec{e}}{\varvec{f}}}^{\mathbf{exp}{\varvec{D}}}}+\left({\mathbf{m}}_{\mathbf{a}\mathbf{u}\mathbf{t}\mathbf{o}\mathbf{v}}-{\mathbf{m}}_{\mathbf{r}\mathbf{o}\mathbf{l}}\right)\frac{{\varvec{d}}{\varvec{v}}}{{\varvec{d}}{\varvec{t}}}+(\mathbf{m}\bullet \mathbf{g}\bullet \mathbf{s}\mathbf{i}\mathbf{n}{\varvec{\upalpha}})$		
Rolling resistance coefficient (KW)	Cf A = ${\mu }_{r}\bullet m\bullet g\bullet v$		
Flexible power coefficient	Cf B = ${\mu }_{w}\bullet m\bullet g\bullet v$		
Air resistance coefficient	Cf C = 0,5 $\bullet \rho \bullet {c}_{w}\bullet {a}_{frontal}\bullet ({v+{v}_{0})}^{2}\bullet v$; (n≈2)		
Engine power extrapolation SIE (SAE J1349)	Ka = ($\frac{990}{p[bar]}$)^1,2^. ($\frac{T[K]}{298}$)^0,6^		
Engine power extrapolation CIE (SAE J1349 si JIS D1001)	Naturally aspirated/turbo Ka = ($\frac{990}{p[bar]}$. ($\frac{T[K]}{298}$)^0,7^)^fm^	40 ≤ $\frac{q}{r}$≤65; fm = 0,036∙ $\frac{q}{r}$ −1.14 $\frac{q}{r}$<40; fm = 0,3 $\frac{q}{r}$>65; fm = 1,2	r = $\frac{{P}_{L}}{{ P}_{E}}$ q = 120,000∙$\frac{F}{D\bullet n}$ (4 stroke)

Depending on both the constructive parameters of the vehicle (weight, height, width, the coefficient of adhesion of the tire to the ground, etc.) as well as the graphic map configured later by the operator, the force that the wheel must overcome when rolling the stand will be calculated:

where

Cf D = air resistance coefficient (with variable n exponential).

exp. D = exponent D(1 ≤ n ≤ 3).

m_autov_ = mass of the vehicle. The reference speed will be 60 km/h for coefficients A and D.

m_rol_ = is the mass of the dynamometer rollers (moment of inertia of the rollers reduced to the translational movement).

v = roller speed.

dv/dt = acceleration of the rollers.

g = gravitational acceleration (9.81 m/s^2^).

α = angle of inclination (±). It is used in the creation of graphic maps from *csv.*

µ_r_ = the adhesion coefficient of the tire with the roller of the dynamometric stand (0.012).

ρ = air density (1.1 kg/m^3^).

a_w_ = air resistance coefficient (0.38).

a_frontal_ = frontal area of the vehicle (l × h).

f_m_ = motor factor (Standard = 0.3).

r = behavior of the pressure in supercharging.

q = specific fuel consumption based on SAE j 1349.

P_L_ = absolute boost pressure.

P_L_ = absolute boost pressure before the turbine (compressor).

F = fuel flow 9(mg/s).

D = cylinder capacity of the engine.

r = rotations per minute of the engine (rpm).

The pollutant emission values are based on the formulas used by the Madur GA 21 Plus model portable analyzer software. They clarify the calculation processes and allow a deeper understanding of the relationships between the variables involved in vehicle emissions. The formulas standardize how emissions are calculated, facilitating comparison between different vehicles or operating conditions otherwise studied in this paper. This allows the performance and efficiency of different vehicle models or emission reduction technologies to be evaluated.

where

CO_2 max_ = characteristic parameter of the fuel used (SIE = 15.4 [%] and CIE = 15.7 [%]).

O_2_ = oxygen content of exhaust gases.

NO = nitrogen oxide (ppm).

O_2ref_ = reference oxygen, conventional parameter, chosen according to the fuel [%],

O_2 meas_ = Oxygen content measured in exhaust gases [%].

20.95 [%] = oxygen concentration in pure air

L = the amount of air entering the engine cylinders at the end of the intake stroke.

L_min_ = the amount of air required to burn one kilogram of fuel.

HC = unburned hydrocarbons.

ICE = internal combustion engine.

SIE = spark ignition engine.

CIE = compression ignition engine.

The combustion process of gasoline or diesel in an ICE requires precise amounts of oxygen to produce combustion. The amount of air required to completely burn a kilogram of fuel varies, depending on the type of fuel and its chemical composition. For complete combustion, a certain amount of oxygen is needed.

For gasoline, the stoichiometric ratio (where λ = 1) between the amount of fuel and the amount of oxygen is approximately 14.7:1 (14.7 kg of air for each kilogram of gasoline). This is the theoretical ratio to ensure complete combustion. For diesel, the stoichiometric ratio is approximately 14.6:1 (14.6 kg of air for each kilogram of gasoline).

It is important to emphasize that in dynamic mode, ICEs do not always reach the ideal stoichiometric conditions due to factors such as the degree of combustion efficiency, the loads to which the engine is subjected being different in a short period of time, and the quality of the air–fuel mixture, factors leading to higher emissions and different air consumption compared to theoretical calculations.

### Experimental design

The research was carried out following two scenarios, each in two phases, focusing on the analysis of emissions of mainly greenhouse gases (CO_2_), nitrogen oxides (NO_x_), and carbon monoxide (CO) from motor vehicles, both petrol and diesel. At the same time, the fuel consumption of these vehicles was also evaluated. According to the first scenario, the main objectives targeted were the collection of real data on the characteristics of road traffic, and the tests were carried out on a judiciously chosen urban route in two phases: the first phase during lunch (congested traffic) and the second phase during the evening hours (light traffic). In the second scenario, the data collected in the two phases on road traffic characteristics are used to generate two driving cycles that simulate real conditions and are used to test any vehicle on the dynamometer; in this case, four different types of vehicles are used (Fig. 2).

### On-board measurement system

Measurements from real traffic are measurements carried out under normal conditions of use, on public roads, in various environments and under various traffic and driving conditions, being directly influenced by factors such as traffic volume, weather and road conditions, driving style, and vehicle load. These measurements are made using PEMS (portable emission measurement system) equipment mounted on moving vehicles or by collecting data while the vehicle is driving on real roads. In this work, such systems are used that record data and store them for later use in the laboratory on the dynamometric stand. Following the collection and processing of these data, test cycles will be designed and later reproduced under controlled and standardized conditions.

**Fig. 1:**
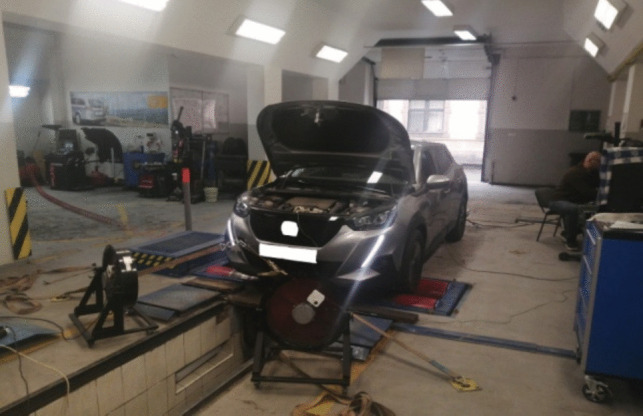
The vehicle positioned on the dynamometer stand during laboratory tests

**Fig. 2:**
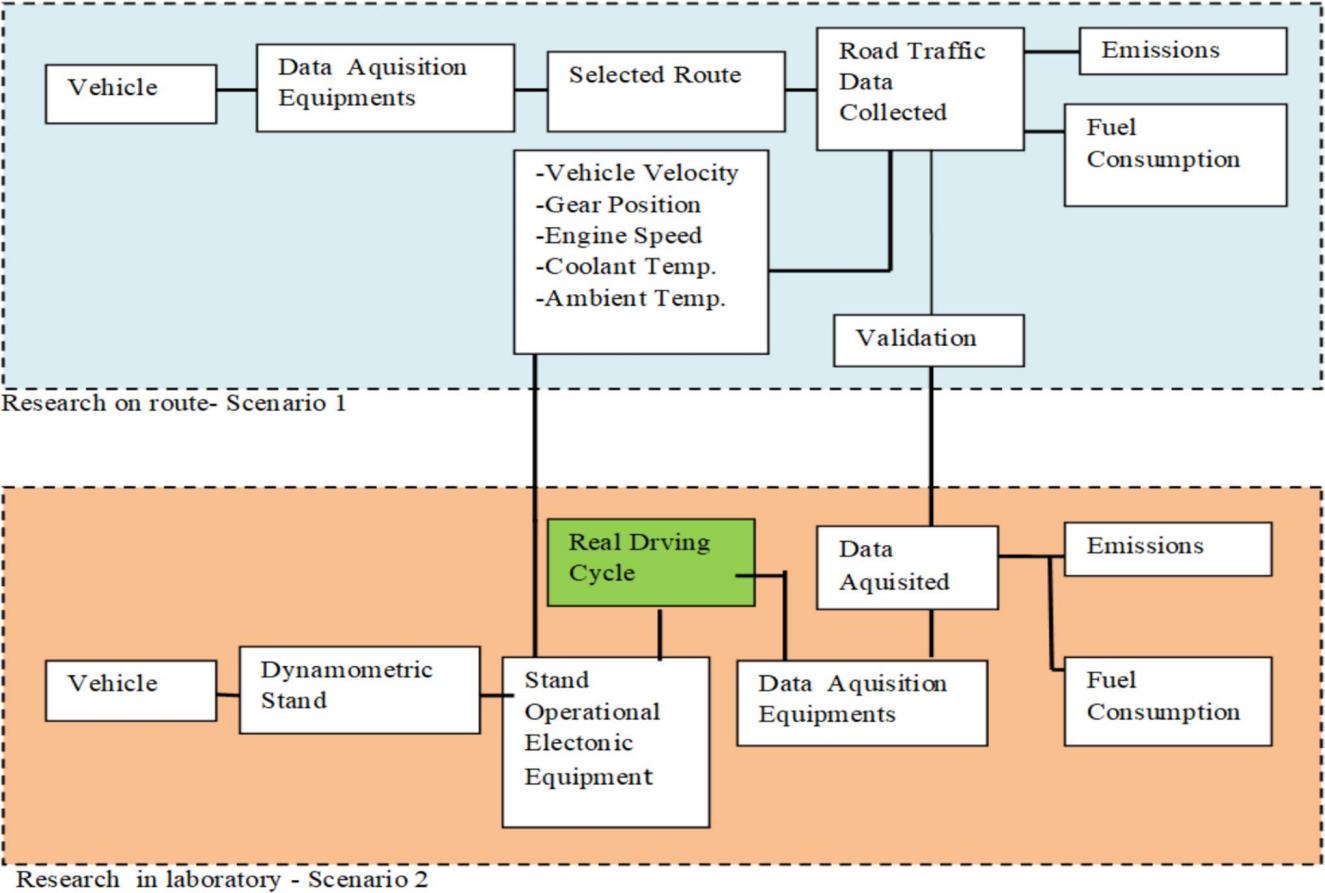
Scheme of research scenarios

During the road tests, the data were recorded with two systems simultaneously, the Madur GA 21 Plus gas analyzer and the Bosch KTS 560 diagnostic system. With the help of the analyzer, emission values such as NO_x_, CO, and CO could be collected (Fig. 1) and the ambient and thermal temperature of the engine, travel speed, time, speed steps, and the value of the calculated load were recorded with the diagnostic software. The fuel consumption was recorded with an AIC 1200 Series fuel flowmeter (it has the option of being connected to the stand) connected to a Board Computer BC 3329 providing finally data in tabular format (Fig. 2).

### Research on dynamometric stand

Following the data collected and recorded during the road tests and processed later, maps were made (in *csv* format) based on speed, time, and gear changes. These maps (real urban driving cycles) are based on the “Driving Cycle” option (found in the dynamometric stand software). The cycles were recreated in a similar way in the laboratory following the route (map) on a screen positioned at the front of the vehicle with the utmost attention.

With the help of these new maps, driving cycles (defensive/offensive driving) can be reproduced in the laboratory in a similar way, thus reaching multiple repetitions and results with the highest possible veracity.

### Research track route

The tests were carried out on a route in the center of Brașov, one of the largest and busiest cities in Romania.

**Fig. 3:**
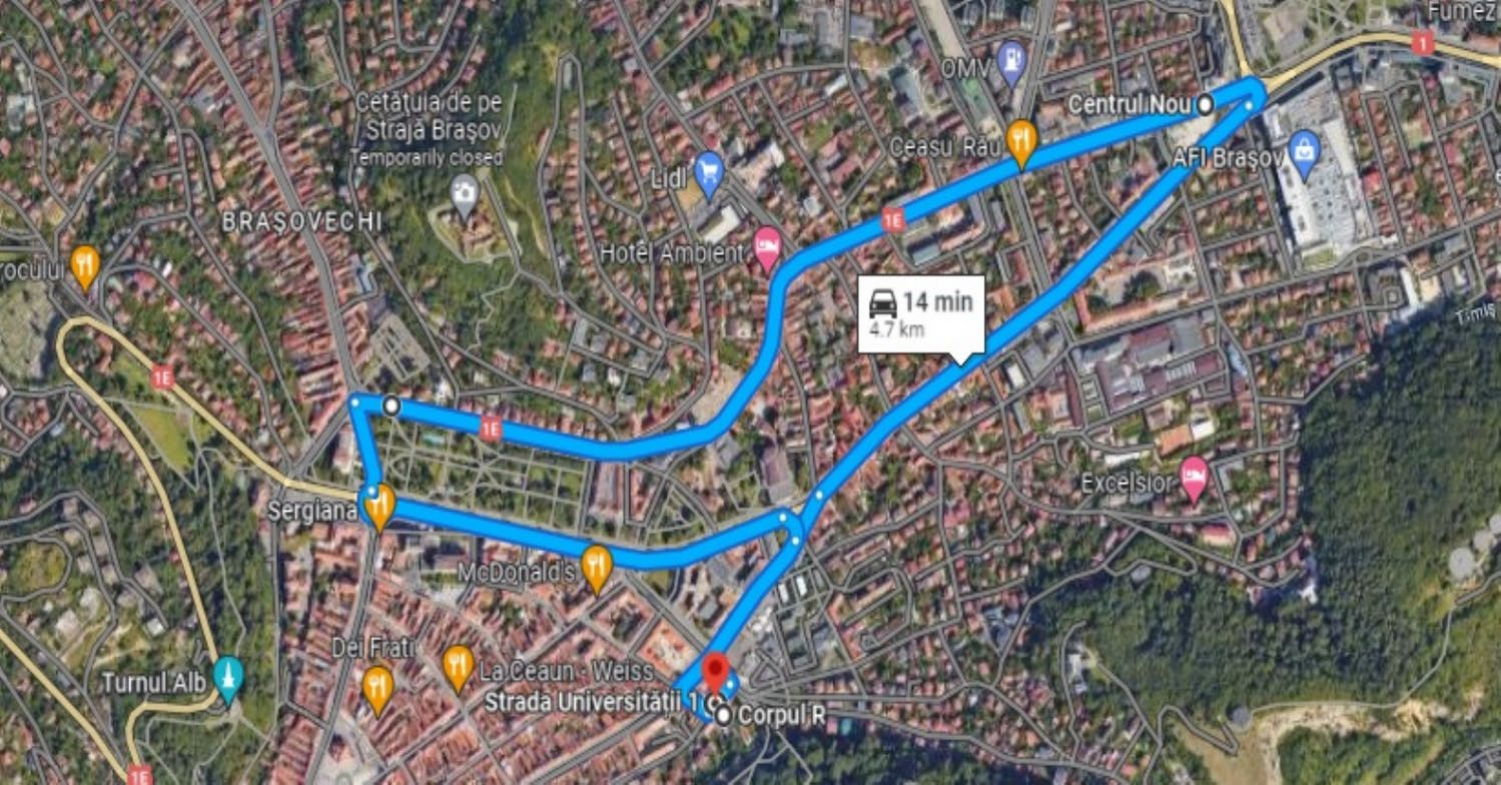
The urban research route (4.7 km) from the centre of the city of Brașov

When selecting the urban route, traffic flows and the incidence of traffic jams along that route were taken into account so that its configuration and road traffic flow (at different times of the day) highlight the problems encountered in real road traffic. Although more than 90% of the route is one-way travel (Table 2), traffic jams did not fail to appear, especially at peak hours 15:00–16:00. The length of the route is approximately 4.7 km (Fig. 3) and the starting point coincides with that of the completion of the urban cycle. In Table 2, one can find the entire route traveled with the exact details of the directions of travel (one-way or two-way).

**Table 2:** Formulas used to calculate vehicle emissions (Cofaru 2007; Grote et al. 2016)

Name of the emissions	Calculation formulas
Carbon dioxide (%)	CO_2_ = CO_2 max_⋅(1- $\frac{{O}_{2}[\%]}{\text{20,95}[\%]}$)
Nitrogen oxides (ppm)	NO_x_[ppm] = $\frac{NO[ppm]}{\text{0,95}}$
Carbon monoxide (relative)	CO_rel_ [mg/m^3^]=$\frac{\text{20,95}\left[\%\right]-{O}_{2ref}}{\text{20,95}\left[\%\right]-{O}_{2meas}}$ ⋅ CO [mg/m^3^]
Carbon monoxide (undiluted)	CO _ned_ = CO ⋅ λ
Coefficient of excess air	λ = $\frac{L}{{L}_{min}}$; λ = $\frac{{CO}_{2}+ \frac{CO}{2}+{O}_{2}+ \left(\frac{\text{1,7261}}{4} \times \frac{\text{3,5}}{\text{3,5}+\frac{CO}{{CO}_{2}}}-\text{0,0088}\right) \times ({CO}_{2}+CO)}{\left(1+\frac{\text{1,7261}}{4}-\text{0,0088}\right)\times \left({CO}_{2}+CO+6 \times HC \times {10}^{-4}\right)}$

Also, the route had to allow studying regulations of pedestrian crossings and stepping between different traffic lights. The research track consisted mostly of main roads and started near the center of Brașov. In order to facilitate the analysis, the track was divided into several Sects. (11), which were defined as either crossroads or connecting pieces between two successive crossroads (Table 3).

**Table 3:** The names of the streets and the direction of movement

Street name	One-way/two way street	Number of lanes
Castelului	One-way street	1
Politehnici	One-way street	1
Nicolae Balcescu	Two-way street	2
15 Noiembrie	One-way street	3–4
Iuliu Maniu	One-way street	2–4
Nicolae Iorga	One-way street	3
Lunga	Two-way street	3
Bulevardul Eroilor	One-way street	3
Nicolae Balcescu	Two-way street	1–2
Dobrogeanu G	Two-way street	2
Castelului	One-way street	1

The intersections with traffic lights (being the ones that take over the highest flow of road traffic) during peak hours (morning and evening) can hardly cope with the large number of vehicles (2800–3750 standard vehicles/hour, Tables 4 and 5). A very important aspect was observed near pedestrian crossings with traffic lights but which are not located in intersections. At lunchtime (when the traffic is not considerable), the number of traffic jams increases in these areas because the traffic engineer cannot intervene and manage the green times for road vehicles, thus directly and negatively influencing polluting emissions and fuel consumption. A similar analysis of road traffic was also analyzed in some traffic lighted intersections on this section, thus reaching the same number of benchmark vehicles (Cofaru et al. 2006).

**Table 4:** The type of intersections, conflict points

The type of intersections and conflict points	Number
Non-traffic crossings for pedestrians	7
Traffic lights for pedestrians	13
Traffic light intersections with light signals	6
Intersections directed by road signals	4
Roundabout circulation	2

**Table 5:** Equivalence coefficients for obtaining standard vehicles

The type of vehicles	Equalization coefficient
Motorcycles (with or without hitch), scooters, etc	0.5
Car (with or without trailer) ≤ 3.5 tons Passenger vehicles up to 18 + 1	1
Vehicles (with or without trailer) ≥ 3.5 tons Passenger vehicles over 18 + 1 (coaches and buses)	2
Articulated bus	4

To establish the equivalence coefficients, it was considered that the standard vehicle (Vt) in the case of cars will be equal to 1 and for articulated buses Vt = 4. Due to the fact that the tests were carried out in an urban cycle, the number of reference vehicles was considerable because the number of articulated buses was high.

### Traffic conditions

The urban track was driven in different traffic conditions: during afternoon (15:00–16:00), the traffic situation is congested, and during evening time (19:00–20:00), when the traffic is fluid, it is a normal situation for this period. The first tests on the road were carried out around afternoon time when urban traffic is at a level compared to peak hours (mornings and evenings). Taking into account the fact that the atmospheric temperature did not exceed 10 °C, and the thermal regime of the vehicles was 30 °C (before the start of the tests), the start & stop function present on some vehicles did not come into operation (taking into account that the function was active on the entire testing period).

It should be mentioned that during all the research on the selected route, the surface of the road infrastructure was dry, and the tire–asphalt adhesion coefficient remains constant.

On the dynamometric stand, the real urban driving cycle is repeated at least 11 times (so that the accuracy of the results is as good as possible).

**Fig. 4:**
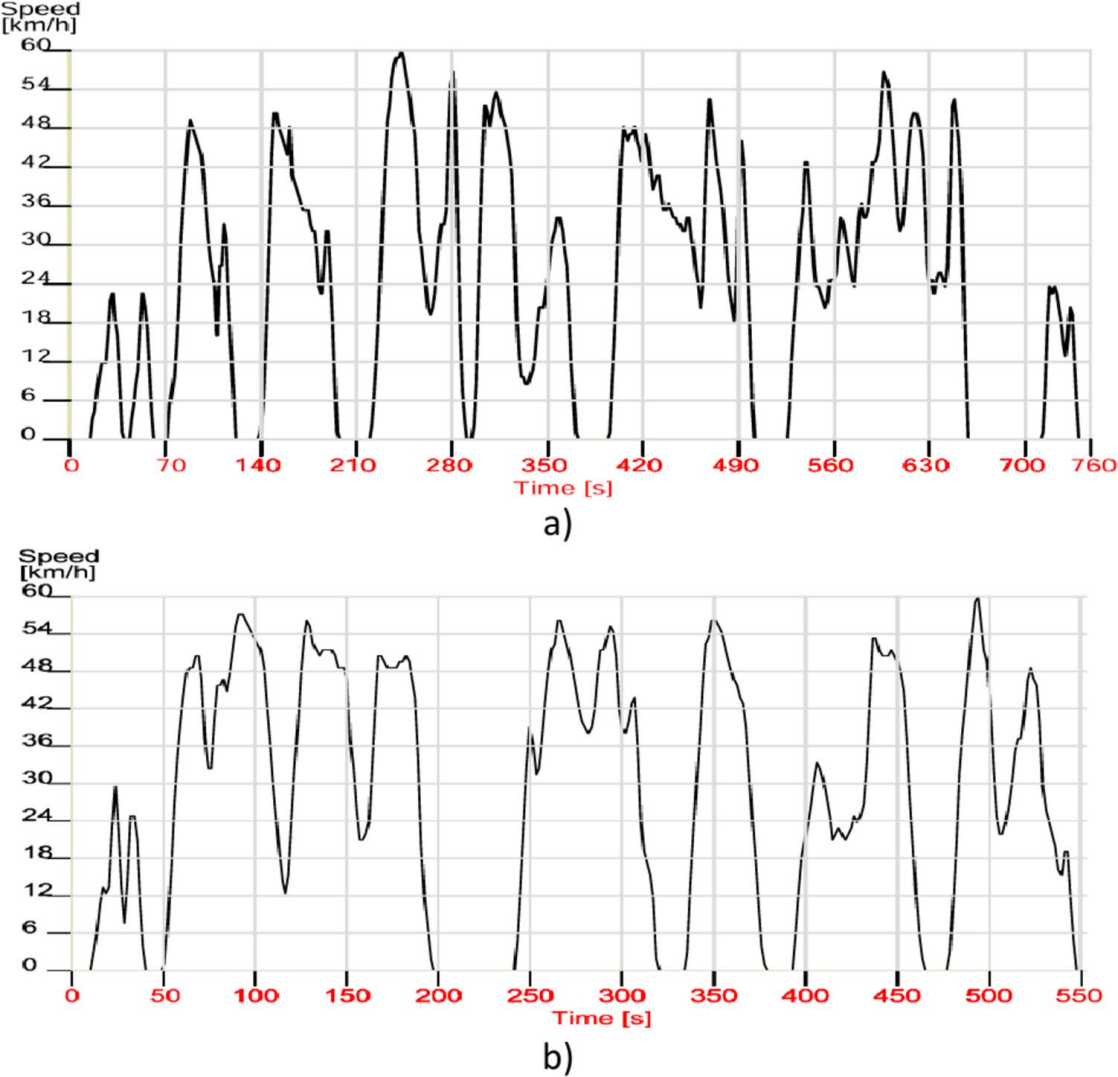
Urban cycle: **a** afternoon; **b** evening (Auto 1-Peugeot)

Figure 4 shows the two real driving cycles in the case of congested traffic (a) and in the case of flowing traffic (b) and Table 6 shows the characteristics of these cycles.

**Table 6:** Real driving cycle characteristic data

Real driving urban cycles		
Cycle characteristics	Afternoon	Evening
Distance [km]	4.7	4.7
Time cycles [s]	450	328
Stop duration [s]	115	69
Number of stops	10	7
Maximum speed [km/h]	56	60
Average speed with stops [km/h]	21	29
Average speed without stops [km/h]	28	37

## Test vehicles

In Table 7, the technical data are specified for each vehicle studied. Vehicle 1 (Start & Stop) and 2 are on gasoline; respectively, 3 and 4 (Start & Stop) are on diesel. Due to the low ambient temperatures and the relatively short test cycles, the Start & Stop function (activated since the beginning of the tests) did not come into operation.

**Table 7.** Technical data of the analyzed vehicles

Car	Vehicle 1 (SIE)	Vehicle 2 (SIE)	Vehicle 3 (CIE)	Vehicle 4 (CIE)
Car model	SUV (bi-turbo) Start & Stop	Sedan	Van (variable geometry turbine)	Sedan (turbo) Start & Stop
Catalyst	Three-way catalytic converter	Three-way catalytic converter	Oxidation catalyst + SCR	Oxidation catalyst + SCR
Displacement [cm^3^], power [HP], gearbox (manual/automatic)	1.2 PureTech (130 HP), Automatic	1798 (142 HP) Manual	1499 (119 HP) Manual	1461 (75 HP) Manual
Mass of the vehicle [kg]	1050	1440	1780	1229

The working mode on the dynamometric stand includes a series of stages to simulate the virtual running of the vehicle on the selected urban route. In the first stage, the data recorded during the actual travel of the selected urban route are entered. The next stage is represented by the generation of the driving cycle. Based on the data entered in the preceding section, the “Driving Cycles” software will generate, through mathematical modeling, the driving cycle of the route selected and traveled in advance. This real driving test cycle is implemented in the stand’s memory and during the research is displayed on the stand’s display. Another stage is represented by connecting the vehicle to the equipment of the dynamometric stand. On this occasion, the constructive data of the vehicle under investigation are entered into the electronic system of the stand, so that during the course of the cycle, the dynamics elements can be calculated to ensure the simulation of the real route recorded.

Each point on the driving cycle curve is marked by a light spot and is tracked in time during the cycle. It should be noted that any point of the driving cycle curve is characterized by the functional parameters of the vehicle’s propulsion system. The connection between the real route traveled by the vehicles and the simulation of traveling these routes through the driving cycle is represented by ensuring the same dynamics of the vehicles. For each vehicle, the forward resistance is simulated based on its own data. These dynamic elements are defined by the presentation materials of the Maha dynamometric stand as coefficients, such as Cf A rolling resistance coefficient (constant), Cf B flexible power coefficient (linear), Cf C air resistance coefficient (*n* ≈ 2), and Cf D air resistance coefficient (with variable n exponential). In fact, these coefficients are expressed in units of its power, representing the components of the vehicle's forward dynamics.

## Results and discussion

The determination of fuel consumption and engine emissions of motor vehicles in road traffic is important for policy making decisions and managing road driving conditions. In urban road networks, traffic control and road characteristics largely determine the driving style through which deceleration and acceleration of cars, which are important factors in fuel consumption and emissions, occur. Traffic control can be optimized to reduce fuel consumption and emissions, which is normally achieved by reducing the number of traffic light stops along the way. Depending on the driving style, drivers react to traffic lights differently: some slow down gradually, while others brake hard only a short distance from the stop line. When starting from a standstill, the acceleration of the vehicle can be smooth or aggressive.

In the research carried out in this study, the driver’s driving style was defensive, respecting the specified legal speed in the urban environment.

Following the tests on the stand using the real driving cycle generated on the basis of the data obtained in the real traffic on the route adopted with the four cars in the two scenarios, traffic at afternoon time (15:00–16:00) and traffic in the evening time (19:00–20:00), data were obtained regarding the instantaneous variation of emissions and vehicle speed.

The results obtained as a result of experimental research aimed at traffic at noon on the chosen route are presented in Fig. 3.

**Fig. 5:**
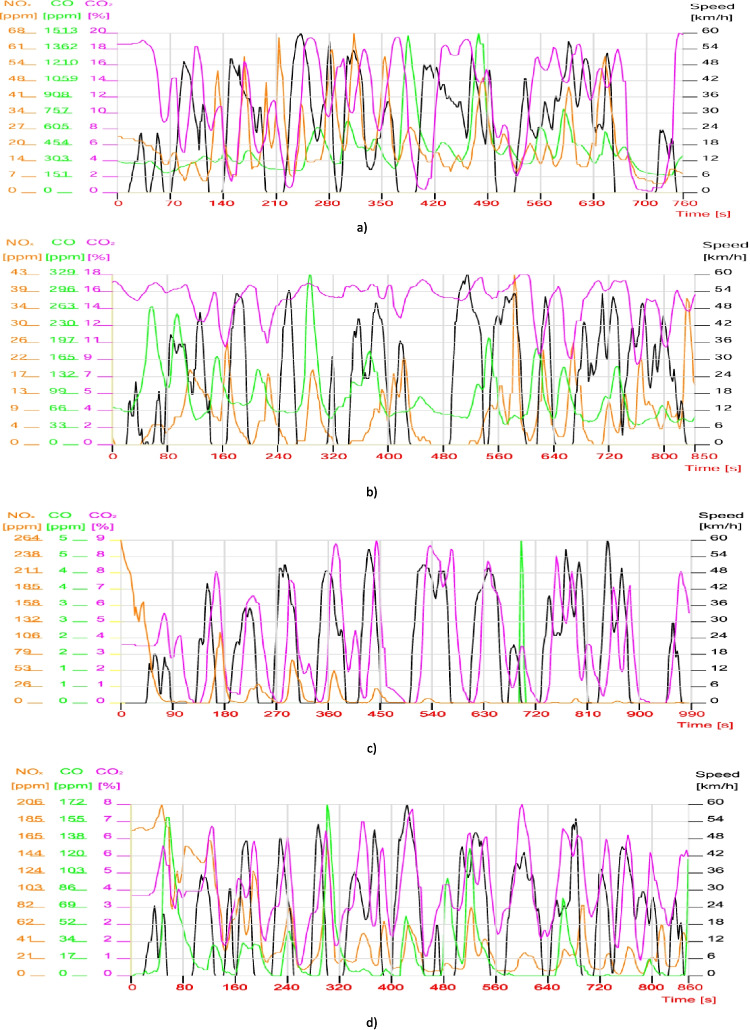
Analysis of vehicle emissions in dynamic urban mode, in the afternoon (15:00–16:00). **A** Vehicle 1, SIE + Three Way Catalyst (TWC); **b** vehicle 2, SIE + Three Way Catalyst (TWC); **c** vehicle 3, CIE + OC + SCR; **d** vehicle 4, CIE + OC + SCR (OC, oxidation catalyst; SCR, selective catalytic reduction)

It is noted that the thermal regime of the vehicles exceeded the atmospheric temperature before the tests began; the analysis of the diagrams shows that NO_x_ emissions do not register high values in the case of gasoline vehicles (Fig. 5a and b). We do not find the same situation in the case of CIEs where the same emissions are recorded since the beginning of the tests with relatively high values (if we make a comparison with those on gasoline), namely NO_x_ = 264 ppm and NO_x_ = 165 ppm (Fig. 5c and d). It should be mentioned that the NO_x_ emissions of the 3-CIE vehicle decrease a lot and remain at extremely low values for a high percentage of the entire urban cycle.

Therefore, if low values of NO_x_ were recorded at SIE, normally CO will have high values as can be seen in Fig. 5a, oscillating sinusoidally depending on the travel speed (300–600 ppm), and Fig. 5b for vehicle 2 where the carbon monoxide varies between 50 and 200 ppm. Similarly, the evolution of CO_2_ has the same tendency, namely to follow the evolution of CO.

In the case of diesel vehicles, the situation is at the opposite pole. Extremely low values were recorded, especially in the case of the third vehicle (variable geometry turbine) where the CO value recorded a peak of 5 ppm. Similarly (but with several peaks over 100 ppm), it is also recorded in the case of the fourth vehicle (with the turbine) where CO varies between 0 and 50 ppm.

The evolution of CO_2_ emissions fluctuates less in the case of SIEs (with an average between 6–16% for vehicle 1 and 11–17% for vehicle 2), while at CIE the percentage of greenhouse gas does not exceed 9%.

**Fig. 6:**
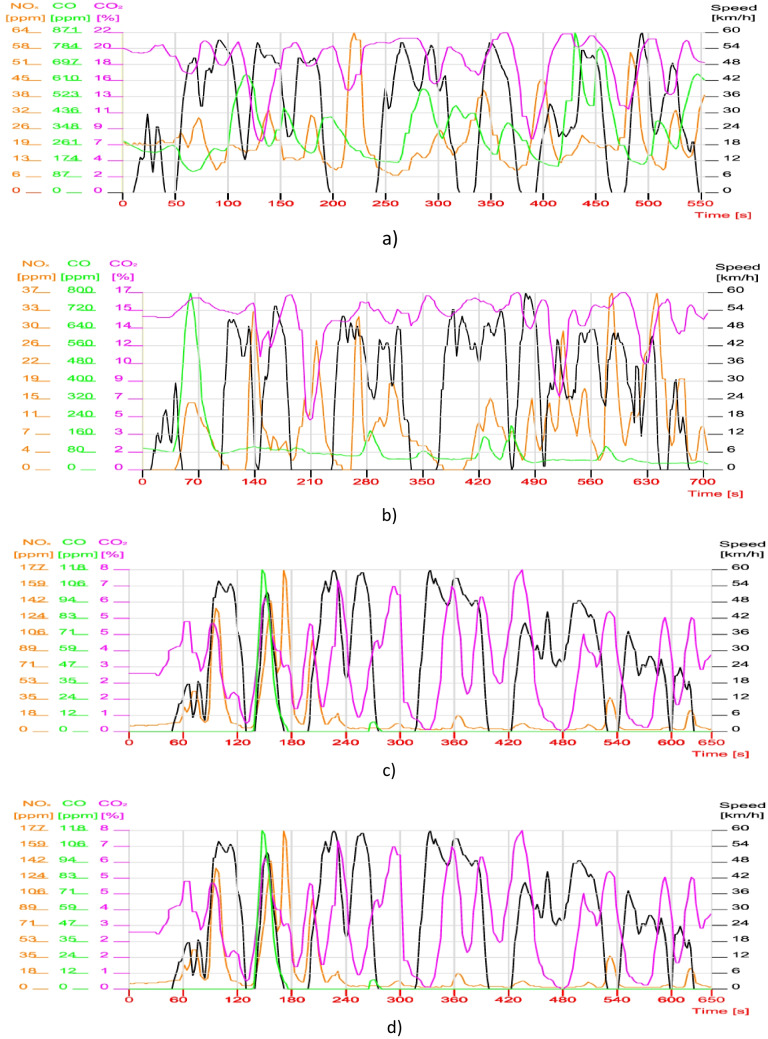
Analysis of vehicle emissions in dynamic urban mode, in the evening. **a** Vehicle 1, SIE + Three Way Catalyst (TWC); **b** vehicle 2, SIE + Three Way Catalyst (TWC); **c** vehicle 3, CIE + OC + SCR; **d** vehicle 4, CIE + OC + SCR (OC, oxidation catalyst; SCR, selective catalytic reduction)

Figure 6 shows the results of the experimental research aimed at the traffic of the four vehicles in the evening (19:00–20:00) on the same route.

In the case of the research that focused on the traffic that took place in the evening (19:00–20:00) for the four vehicles, it was found that the average speed increased slightly (V_med_ = 29.0). Although the traffic flow is lighter, NO_x_ emissions tend to have the same values both at SIE and at CIE with the specification that in the case of diesel vehicles these emissions no longer start from very high values, as shown in Fig. 6c and d, but from around the values of NO_x_ = 65 ppm for vehicle 3 and NO_x_ = 90 ppm for vehicle 4 (with the specification that the thermal regime of the engines before departure increased by 12 °C at CIE). Greater attention was paid to vehicle 3 where the NOx values show a significant decrease with a tendency to level out in both test cycles (below 20 ppm) (Diminig et al. 2022; Yuhua et al. 2023).

The evolution of CO emissions at SIE is largely the same in both cycles (disregarding the few peaks). A slight improvement can be observed in vehicle 2 SIE (Fig. 6b) where the CO values tend towards a uniformity with a tendency to decrease below 80 ppm.

The CO_2_ emission variations do not significantly change their oscillatory variations during the two operating cycles, the peaks continuing to be similarly influenced by accelerations and decelerations.

The vehicles used for the tests are provided with exhaust gas control and treatment systems according to EURO 6 pollution standards, and these cover the types and classes of light vehicles that circulate in Brașov.

In the analyzed cases, the parameter that shows the influences of the various road traffic constraints (distances between conflict points, speed limits, congestion, etc.) is the velocity of the vehicle. This travel velocity has a continuous variation both in the tests on the road for the acquisition of data and on the dynamometric stand due to the course of the cycle that must be followed and which will determine a transient operation of the engine of the propulsion system. These transient operating regimes of the engines will influence the energy and ecological parameters of the vehicles. If we refer to nitrogen oxides, then both their formation and the possibility of their reduction with the available treatment systems (TWC system for SIE or SCR system for CIE) will be influenced by these transient regimes as: acceleration, deceleration, and going to constantly under load or idling (Orkun et al. 2022).

The temperature level of the gases in the combustion chamber is the essential influencing parameter of the NO_x_ formation reactions.

The factors that decisively influence the formation mechanisms of NO_x_ emissions in internal combustion engines and that manifest themselves in ways specific to the type of engine (SIE or CIE) are as follows: the quality of the mixture given by the air–fuel ratio, in the sense of increasing excess air the mixture causes the combustion temperature to decrease; the fraction of burnt gases (residual and/or recirculated) present in the fresh air–fuel mixture in the combustion chamber and which has an effect similar to the previous case; and the amount of fuel–air mixture that burns before the piston reaches top dead center.

The functional parameters of the engine, such as the speed and the load at a given moment, have a major influence on the temperature of the combustion gases by changing the mentioned factors, which, by increasing or decreasing it, will determine larger or smaller amounts of nitrogen oxides formed.

**Fig. 7:**
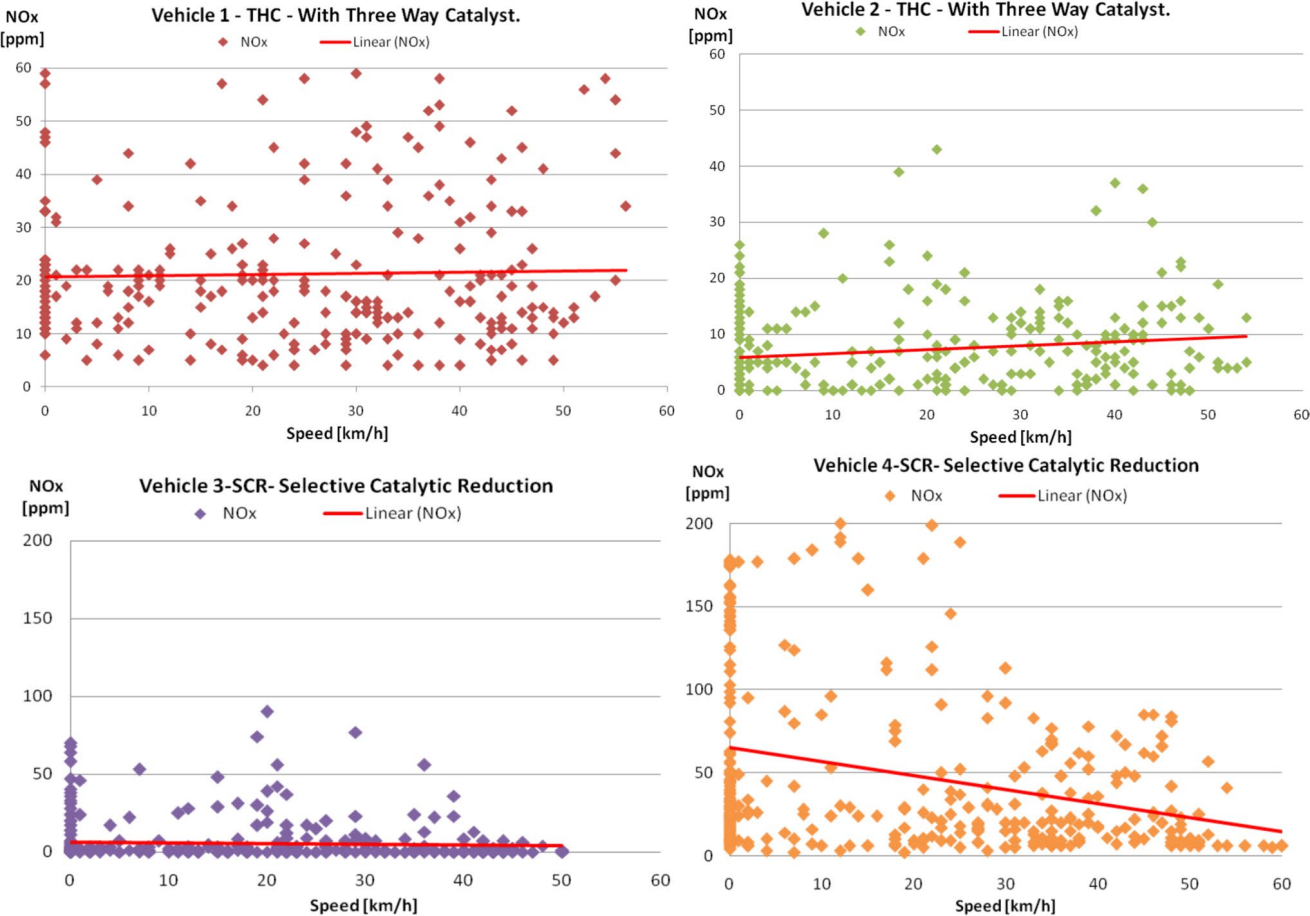
NO_x_ distribution depending on the speed of the vehicle (afternoon time)

**Fig. 8:**
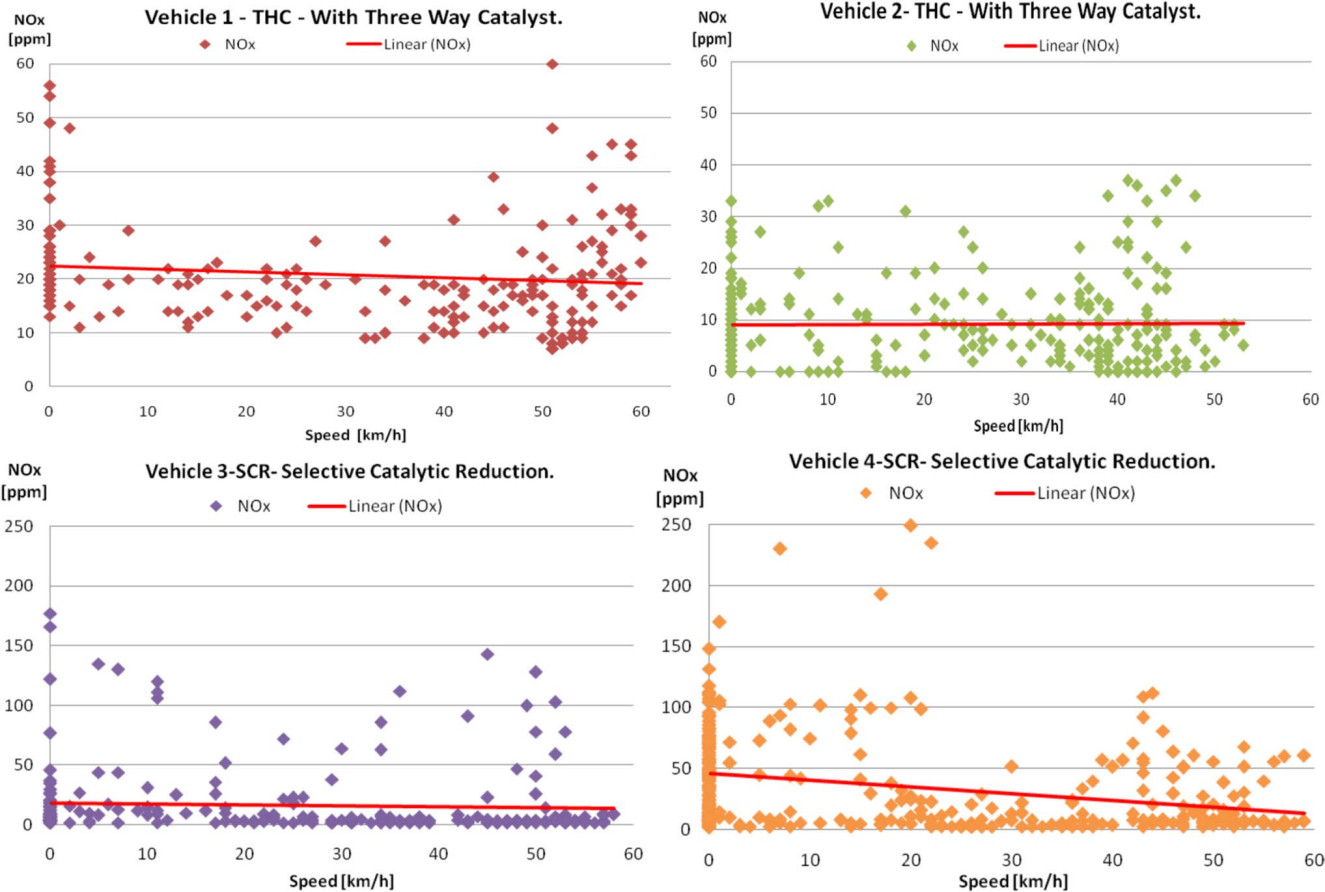
NO_x_ distribution depending on the speed of the vehicle (evening time)

The collected data regarding the instantaneous NO_x_ emissions depending on the vehicle velocity for the two traffic conditions are presented in Figs. 7 and 8.

It is mentioned that the velocity of the vehicles varies between the values of 0 and over 50 km/h. The value of the travel velocity of 0 km/h means that the vehicle is stationary without the engine being stopped, and the emissions are measured.

A certain travel velocity can be achieved with different engine speeds and loads that will generate different emissions; this is how it is explained that, for a certain travel speed, different emission values are recorded. In addition, exhaust gas treatment systems have different pollutant conversion rates for different gas flow rates for the same travel velocity. This can be the explanation for the spread of points representing the measured instantaneous values of the NO_x_ emission depending on the travel velocities.

In congested traffic conditions, the variation of nitrogen oxides shows a slight increase, applying a linear regression to the data in SIE vehicles (Fig. 7, vehicles 1 and 2) with the increase in travel speed, which implies an increase in engine load, which causes an increase in combustion temperatures and an increase in the rate of NO_x_ formation, and the three-way converter has an efficiency limited by the operating temperature (Mahadevan and Subramanian 2017), while at CIE, the trend is the opposite (Fig. 7 vehicles 3 and 4); i.e., they tend to decrease with increasing vehicle speed; this evolution can be attributed to the higher efficiency of the SCR system.

It is noted that both SIE and CIE have higher NOx values when starting the engine cold (Fig. 7). This is primarily explained by the fact that the NO_x_ treatment systems have not reached the level to start the NO_x_ reduction process (light-off temperature). The light-off temperature for the three-way catalyst (TWC) is between 250 and 300 °C depending on the construction characteristics, and for the selective catalytic reduction system (SCR) around 200 °C.

In addition, the analysis of NO_x_ emissions must take into account the effects given by the temperature level of the air admitted into the engine, which influences the maximum value of the cycle temperature and therefore the NO_x_ emissions, and the recirculation of burnt gases (internal or external EGR) in the supercharged engine leads to the simultaneous reduction of NO_x_ and HC (Cofaru 2012; Xin et al. 2019).

In the case of the evening urban cycle (Fig. 8), where the thermal regime of the engine is already approaching an optimal engine operation, a rarefaction or decrease of NO_x_ can be observed at certain travel speeds, namely vehicles 1 and 2 (between 30–40 km/h and 20–35 km/h, respectively) and vehicles 3 and 4 (between 30–45 km/h and 20–40 km/h, respectively). We cannot notice this even in the case of the urban cycle after noon, where the thermal regime of the engine was lower.

Looking at the NO_x_ values and comparing the different urban cycles, it is found that in the evening cycle, some values increase slightly both at SIE and at CIE, but without influencing in any way the tendency of linear decrease depending on the travel speed which otherwise remains evident in the case of CIEs. The NO_x_ values can increase above the equilibrium values when the temperature decreases during the expansion and the peak of the NO_x_ concentration, which appears in the expansion, is influenced by the moment of initiation of the ignition of the dosage (fuel mixture), respectively the operating conditions of the engine (Cofaru 2015; Muhamad and Ocktaek 2022).

**Fig. 9:**
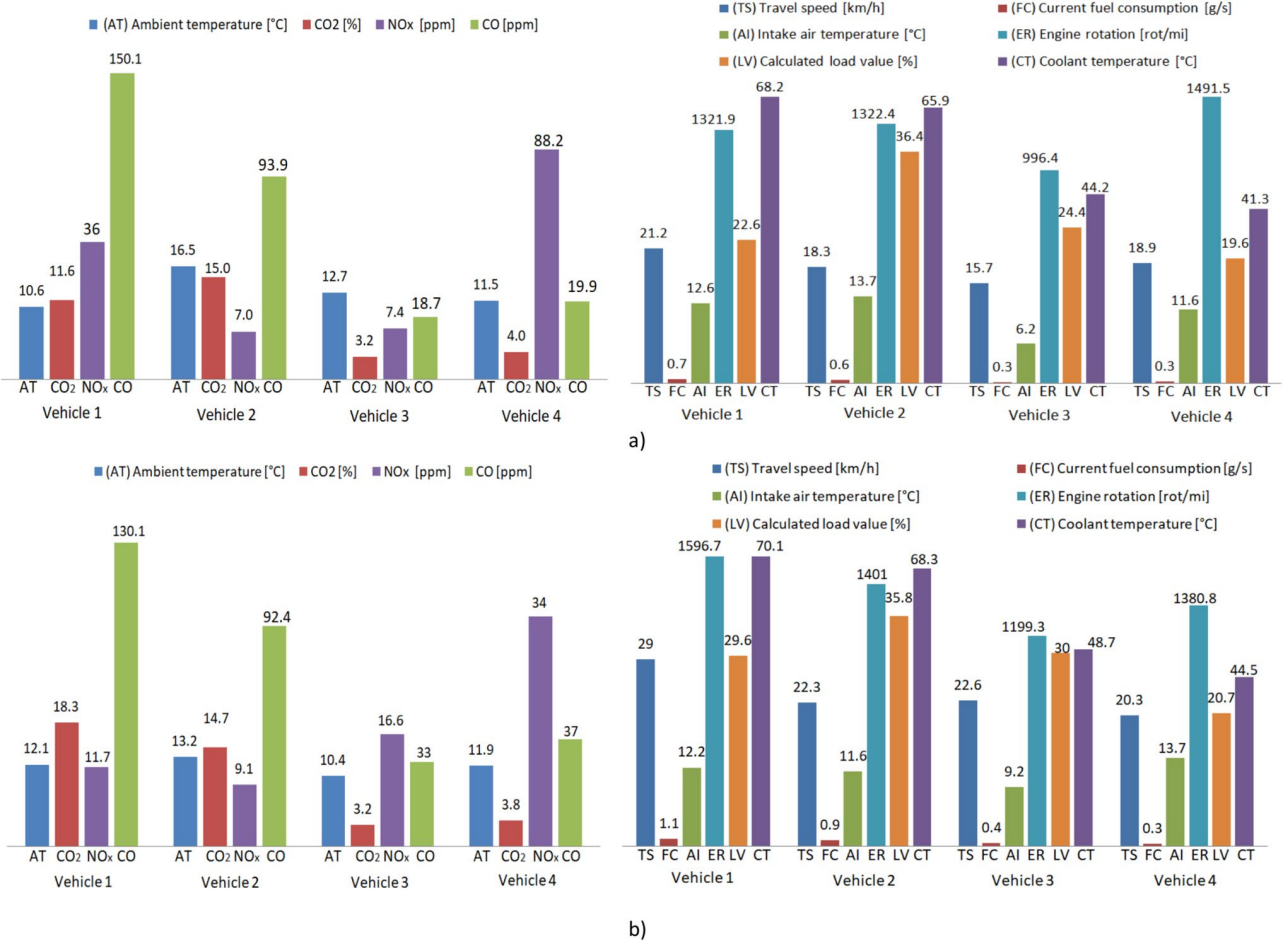
Analysis of average values of vehicle emissions, fuel consumption, and other parameters. **a** Afternoon cycle, **b** evening cycle

Interesting conclusions can also be obtained based on the analysis of the average values of emissions, fuel consumption, and other functional parameters presented in Fig. 9. During the afternoon urban cycle, the average values of NO_x_ emissions are close for vehicle 2 SIE (7 ppm) and vehicle 3 CIE (7.4 ppm) and a significant difference exists between vehicle 1 SIE (36 ppm) and vehicle 4 (88.2 ppm). It should be noted that the previously mentioned vehicles have increases and decreases, respectively, in the evening urban cycle, especially in the case of vehicle 1 and vehicle 4, where the NO_x_ values decrease by 3 times.

The values of CO emissions remain at high values in both test cycles, with the clear specification that the highest values are found in gasoline vehicles.

Compared to fuel consumption, gasoline vehicles (vehicle 1 ≈ 0.7–1.1 g/s and vehicle 2 ≈ 0.6–0.9 g/s) register significantly higher values than diesel ones (vehicle 3 ≈ 0.3–0.4 g/s and the vehicle 4≈0.3 g/s) under the conditions where the calculated load has higher values at SIE compared to CIE, V_mean_ = 21.0 km/h (in the afternoon) and V_mean_ = 29.0 km/h (in the evening) and the average atmospheric temperature is 12.3 °C.

There is a slight difference in fuel consumption between vehicles, especially in the evening cycle where vehicle 1 (SIE) consumes more by 14%, in the conditions where the calculated load is inversely proportional and vehicle 3 (CIE) by up to 21%.

## Conclusions

The research aimed at determining the influence of road and traffic conditions on emissions and fuel consumption of light vehicles in a real urban driving cycle or was carried out both on the urban route chosen for tests and on the dynamometric stand following a cycle built on the basis of data collected on the real circuit.

From the analysis of the obtained results, the following main conclusions can be formulated:

- The research route was chosen specifically to describe the existing street organization, i.e., portions of the urban network without traffic control and management, signalized pedestrian crossings but not synchronized with the other signaling systems, pedestrian crossings without light signaling, signalized intersections with traffic lights, and roundabouts. All these elements determined the sectioning of the traffic flow causing frequent decelerations and accelerations that lead to increases in fuel consumption and emissions. From these results, the need to improve the control and integrated traffic management of the entire urban street network emerges.
- From the point of view of the vehicles, they presented fuel consumption and emissions influenced primarily by the particularities of the constructive parameters and depended on the thermal regime of the engine. In the case of the study, it was observed that the low thermal regime (15–30 °C) in the case of CIE vehicles negatively influences NO_x_ emissions when high values are recorded (especially at start-up) and low values in the case of CO emissions, and for SIE the situation, it is presented the other way around. Along with the increase in the thermal regime of the engine both at SIE and at CIE, nitrogen oxides show a percentage decrease between certain travel speeds, especially at CIE: vehicle 3 between 30 and 45 km/h and vehicle 4 between 20 and 40 km/h and above 40 km/h, NO_x_ registers increasing values.
- The efficiency of gas treatment equipment is influenced by the temperature of the exhaust gases.
- The results of experimental research showed differences when urban traffic registers increased traffic flows, thus leading to polluting emissions and high fuel consumption. In the case of congested traffic, frequent stops, standstills, accelerations/decelerations (transient regime), low ambient temperatures (6–10 °C), the thermal regime of the engines being below 45 °C (CIE) and 70 °C (SIE), and very low travel speeds (Vmean = 18.5–23.5 km/h) led to high values of pollutant emissions and fuel consumption.
- The driver’s behavior and manner of driving are the key elements in urban traffic situations where accelerations and decelerations can be executed smoothly or aggressively. Under these proper circumstances, the factor long-term education programs through media are demanded to understand the close relationship between environmental requirements and the way of driving a vehicle on public roads.

## Data Availability

The datasets used and/or analyzed during the current study are available from the corresponding author on reasonable request.

## Abbreviations

v: Vehicle velocity (km/h)
CO: Carbon monoxide (ppm)
NO_x_: Nitrogen oxides (ppm)
HC: Hydrocarbons
CO_2_: Carbon dioxide (%)
SIE: Spark ignition engine
CIE: Compression ignition engine
OC: Oxidation catalyst
V_t_: Reference vehicle (vehicles/hour)
SCR: Selective catalytic reduction
Cf A: Rolling resistance coefficient (KW)
Cf B: Flexible power coefficient
Cf C: Air resistance coefficient
WLTP: Worldwide harmonized light-duty vehicles test procedure
OBD: On-board diagnostic
PEMS: Portable emission measurement system
